# Improving Pediatric Neuro-Oncology Survival Disparities in the United States–Mexico Border Region: A Cross-Border Initiative Between San Diego, California, and Tijuana, Mexico

**DOI:** 10.1200/GO.20.00377

**Published:** 2020-11-20

**Authors:** Paula Aristizabal, Luke P. Burns, Nikhil V. Kumar, Bianca P. Perdomo, Rebeca Rivera-Gomez, Mario A. Ornelas, David Gonda, Denise Malicki, Courtney D. Thornburg, William Roberts, Michael L. Levy, John R. Crawford

**Affiliations:** ^1^ Peckham Center for Cancer and Blood Disorders, Rady Children’s Hospital San Diego, San Diego, CA; ^2^ Division of Pediatric Hematology/Oncology, Department of Pediatrics, University of California, San Diego, La Jolla, CA; ^3^ Population Sciences, Disparities and Community Engagement, Moores Cancer Center, University of California, San Diego, La Jolla, CA; ^4^ School of Medicine, University of California, San Diego, La Jolla, CA; ^5^ Hospital General de Tijuana/Universidad Autónoma de Baja California Tijuana, Baja California, Mexico; ^6^ Department of Neurosciences, University of California, San Diego, La Jolla, CA; ^7^ Division of Pediatric Neurosurgery, Department of Neurosurgery, University of California, San Diego, La Jolla, CA; ^8^ Department of Pathology, University of California, San Diego, La Jolla, CA; ^9^ Division of Pediatric Neurology, Department of Pediatrics, University of California San Diego, La Jolla, CA

## Abstract

**PURPOSE:**

Treatment of children with CNS tumors (CNSTs) demands a complex, interdisciplinary approach that is rarely available in low- and middle-income countries. We established the Cross-Border Neuro-Oncology Program (CBNP) between Rady Children’s Hospital, San Diego (RCHSD), and Hospital General, Tijuana (HGT), Mexico, to provide access to neuro-oncology care, including neurosurgic services, for children with CNSTs diagnosed at HGT. Our purpose was to assess the feasibility of the CBNP across the United States-Mexico border and improve survival for children with CNSTs at HGT by implementing the CBNP.

**PATIENTS AND METHODS:**

We prospectively assessed clinicopathologic profiles, the extent of resection, progression-free survival, and overall survival (OS) in children with CNSTs at HGT from 2010 to 2017.

**RESULTS:**

Sixty patients with CNSTs participated in the CBNP during the study period. The most common diagnoses were low-grade glioma (24.5%) and medulloblastoma (22.4%). Of patients who were eligible for surgery, 49 underwent resection at RCHSD and returned to HGT for collaborative management. Gross total resection was achieved in 78% of cases at RCHSD compared with 0% at HGT (*P* < .001) and was a predictor of 5-year OS (hazard ratio, 0.250; 95% CI, 0.067 to 0.934; *P* = .024). Five-year OS improved from 0% before 2010 to 52% in 2017.

**CONCLUSION:**

The CBNP facilitated access to complex neuro-oncology care for underserved children in Mexico through binational exchanges of resources and expertise. Survival for patients in the CBNP dramatically improved. Gross total resection at RCHSD was associated with higher OS, highlighting the critical role of experienced neurosurgeons in the treatment of CNSTs. The CBNP model offers an attractive alternative for children with CNSTs in low- and middle-income countries who require complex neuro-oncology care, particularly those in close proximity to institutions in high-income countries with extensive neuro-oncology expertise.

## INTRODUCTION

Pediatric CNS tumors (CNSTs) remain one of the most challenging tumors to treat in low- and middle-income countries (LMICs)^1^ as a result of significant deficits in infrastructure, human resources, evidence-based treatments, and interdisciplinary teams trained in neuro-oncology.^2-5^ CNSTs comprise 15%-20% of all childhood neoplasms, are the second most common childhood malignancy after leukemia, and the first cause of mortality in children with cancer. However, after recent improvements in surgical interventions, imaging studies, and histopathologic classification systems, 5-year progression-free survival (PFS) for children with CNSTs in high-income countries (HICs) is as high as 70%-80%.^1,3-7^ Unfortunately, in LMICs, where 80% of the world’s children reside, 5-year overall survival (OS) for children with CNSTs is 0%-40%.^1-5,8,9^ Delivery of effective therapy for children with CNSTs demands a multimodal management of surgery, chemotherapy, and/or radiotherapy depending on the diagnosis,^6^ and poses a particular challenge in LMICs, such as Mexico, given the need for a comprehensive, interdisciplinary approach. This includes timely access to sophisticated neurosurgical and intensive care with experienced neuro-oncologists, pediatric neurosurgeons, neuro-radiologists, radiation oncologists, neuro-pathologists, and intensivists, which is frequently absent in LMICs.^10,11^ To deliver effective therapy for children with CNSTs in Mexico, we established the Cross-Border Neuro-Oncology Program (CBNP) in 2010 between the Hospital General, Tijuana (HGT), and Rady Children’s Hospital, San Diego (RCHSD), located 20 miles from HGT.

**CONTEXT**

**Key Objective**
The Cross-Border Neuro-Oncology Program was established across the United States-Mexico border to facilitate access to neuro-oncology care for children with CNS tumors (CNSTs) in Tijuana, Mexico, through binational exchanges of resources and expertise.
**Knowledge Generated**
Survival for patients in the Cross-Border Neuro-Oncology Program dramatically improved. Gross total resection through the partnership was associated with higher overall survival.
**Relevance**
The cross-border model provides complex neuro-oncology care to underserved children and serves as a feasible model for other border regions. Mentored neuro-oncology care of children with CNSTs vastly improved survival at Hospital General, Tijuana. Our model offers a suitable option for children with CNSTs in low- and middle-income countries who require surgical resection, particularly those in close proximity to high-income institutions capable of offering these complex services.

Our purpose was to assess the feasibility of the CBNP across the United States-Mexico border and improve survival for children with CNSTs at HGT. We leveraged the already established twinning program between RCHSD and HGT,^12,13^ and we provided comprehensive neuro-oncology care, including access to high-quality neurosurgical services at RCHSD, and targeted training and infrastructure enhancement at HGT. In this manuscript, we describe the implementation of the CBNP and clinical outcomes of patients who participated in the CBNP.

## PATIENTS AND METHODS

### CBNP Implementation

Mexico is one of the most populous countries in the world, with high poverty rates (41.9% in 2018)^14^ and profound socioeconomic disparities, including a Gini index of 45.4 (7th highest in the region, 2018).^15^ (The Gini index is a measure of statistical dispersion intended to represent the income or wealth distribution of a nation’s residents, and is the most commonly used measurement of inequality.)

The border Mexican state of Baja California had 1 million children in 2015, 50% of whom were uninsured^16,17^ (Table 1). Tijuana is the 6th most populous city in Mexico^16^ and shares a 24-km border with San Diego (2nd largest Californian city and 8th largest in the United States).^18^ Sixty-eight million people cross this border annually, making it the world’s most transited border.^19^ There are vast cross-border health disparities^14-18^ (Table 1), which contribute to survival gaps in children with cancer.^12,13,20^

**TABLE 1:**
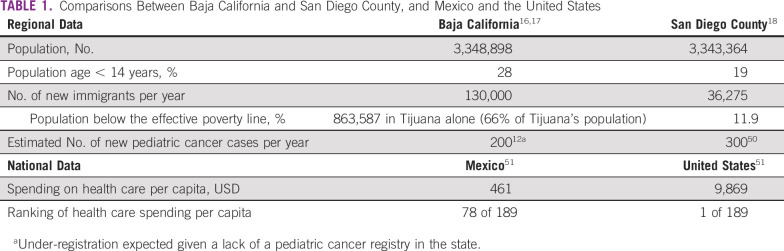
Comparisons Between Baja California and San Diego County, and Mexico and the United States

**FIG 1:**
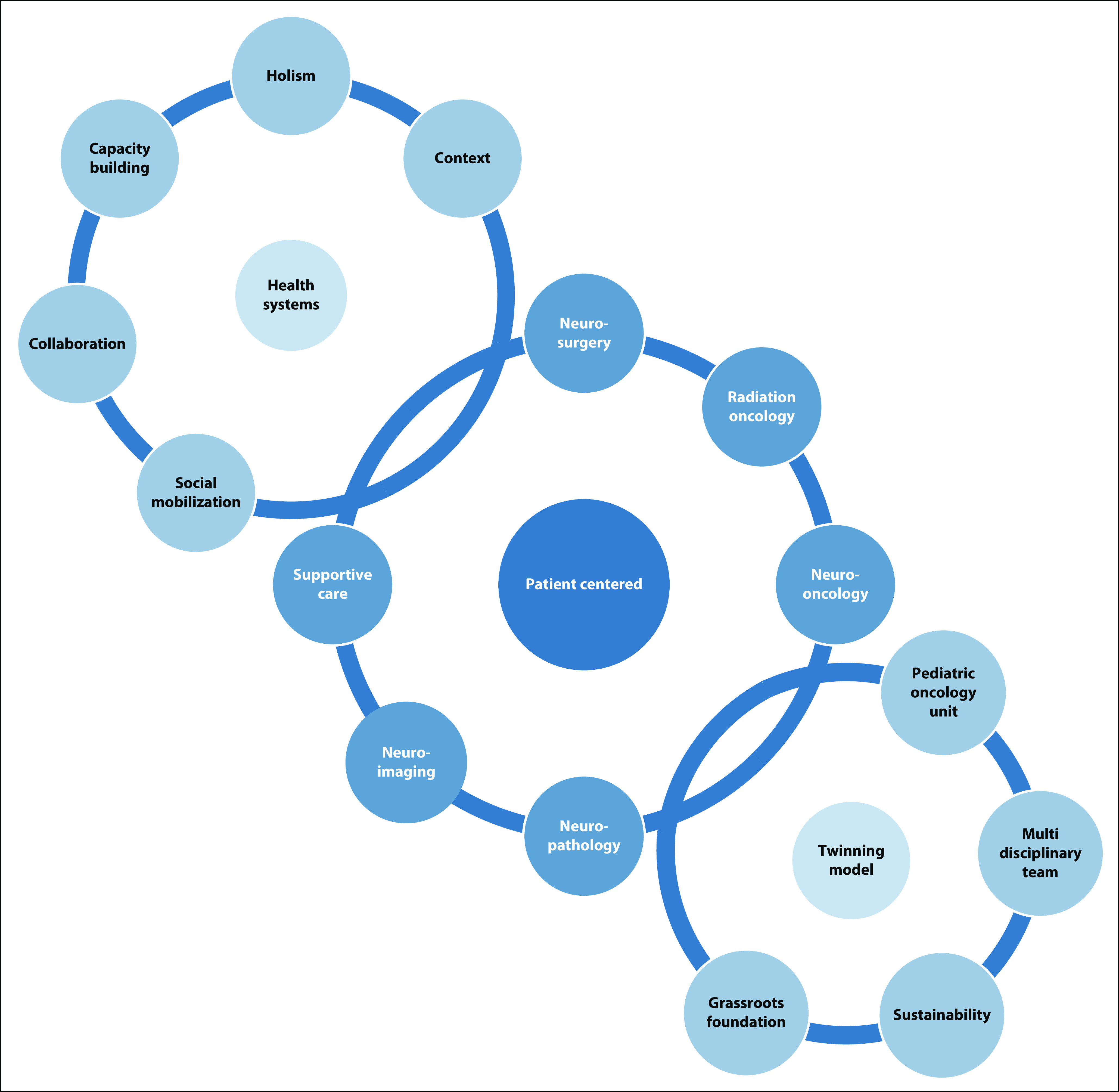
Cross-Border Neuro-Oncology Program and collaboration model between Rady Children’s Hospital, San Diego, and Hospital General, Tijuana, based on the Swanson’s health systems, twinning, and patient-centered neuro-oncology models.

To address these disparities, we established a twinning program^21^ in 2008 between RCHSD, St Jude Children’s Research Hospital, and HGT to improve access to care and outcomes in children with cancer in Tijuana.^12,13^ Using this twinning program as a platform, we designed our CBNP model (Fig 1) by implementing key components of:

- Twinning in pediatric neuro-oncology, including telemedicine^22-24^
- Health system strengthening principles^12,25^
- Patient-centered care
- The global neurosurgery initiative from the 2015 Lancet Commission on Global Surgery.^26-28^

We adapted a needs assessment tool^29^ to identify key requirements for a neuro-oncology program.^29^ The needs assessment, completed in 2009, revealed that, although HGT had basic oncology, nursing, imaging, and radiotherapy services, it did not have neurosurgical equipment, experienced neurosurgeons, or a specialized interdisciplinary team to provide comprehensive neuro-oncology care.

Similar to barriers reported in other LMICs, we also found inadequate pediatric neuro-oncology training for health care providers, delays in diagnosis and radiotherapy, deficiencies in referral and diagnostic pathways, and limited access to accurate diagnosis and chemotherapy protocols.^2,9,30,31^ All patients with CNSTs (n = 16) diagnosed before CBNP implementation (2002-2009) died (data from HGT hospital-based cancer registry, unpublished).

By leveraging the unique characteristics of HGT and its close proximity to RCHSD, we developed an action plan that included (1) a patient evaluation and transfer workflow at HGT and RCHSD, and (2) targeted training and infrastructure enhancement at HGT, such that patients with CNSTs that required surgical resection could be transferred from RCHSD back to HGT for additional neuro-oncology management.

### Study Population

**FIG 2:**
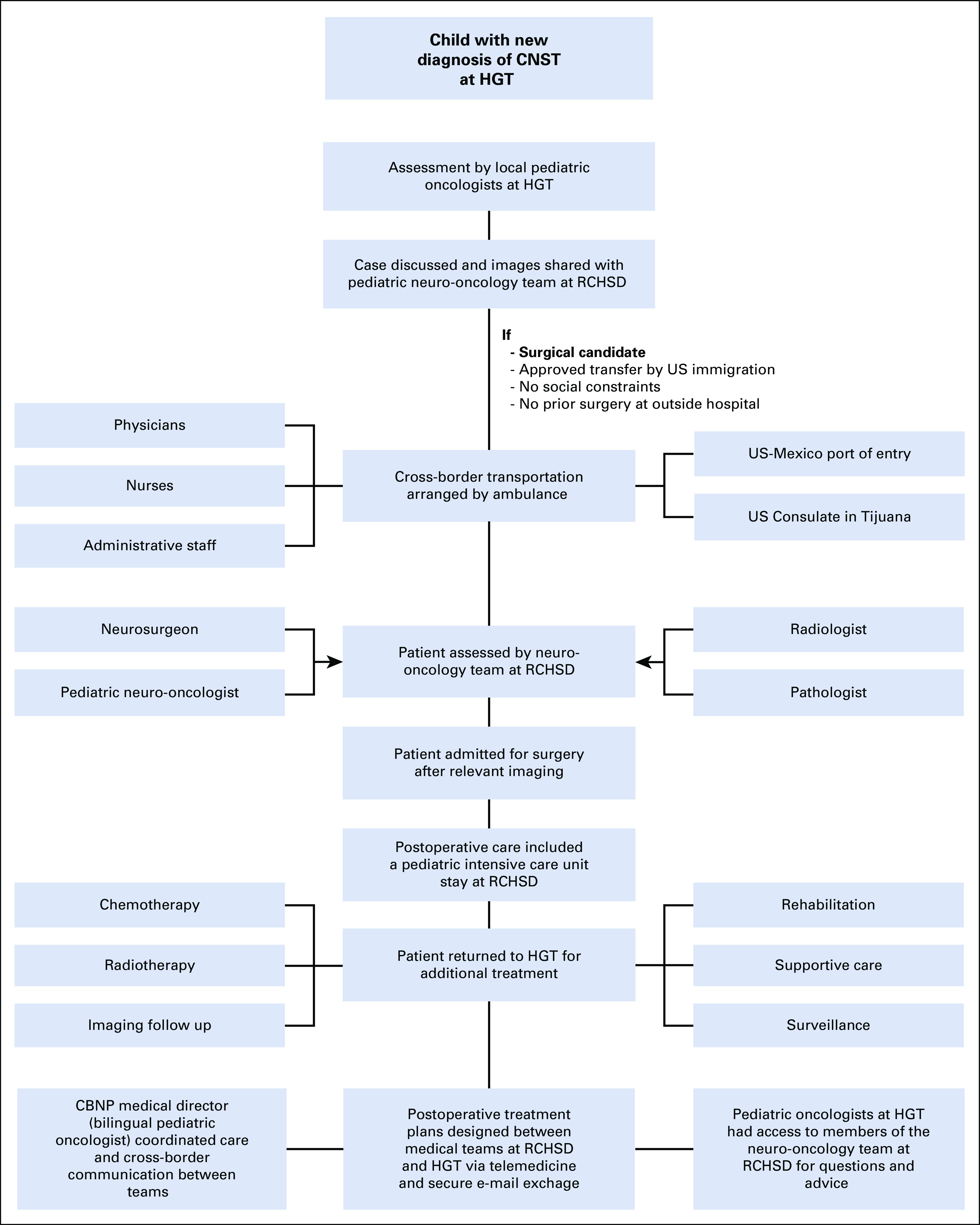
Workflow for patients participating in the Cross-Border Neuro-Oncology Program (CBNP) from diagnosis to collaborative treatment. CNST, CNS tumor; HGT, Hospital General, Tijuana; RCHSD, Rady Children’s Hospital, San Diego.

We used the HGT registry database to prospectively identify eligible patients. Patients age 0-21 years were eligible to participate in the CBNP if they were diagnosed with a CNST amenable for neuro-surgical intervention between January 1, 2010 and December 31, 2017, and had not yet undergone surgical resection (Fig 2 and Appendix). Sixty-five patients were diagnosed with CNSTs during the study period. Five patients were excluded from the cohort for the following reasons: diagnosis of brainstem glioma after biopsy (n = 3), lost to follow up (n = 1), and diagnosis of a vascular malformation (n = 1). We defined being lost to follow up as a patient missing a scheduled appointment without medical justification during active therapy (treatment abandonment) or after finishing therapy and being noncontactable for 6 months. Our final cohort included 60 Mexican patients. The institutional review boards for the University of California San Diego/RCHSD and HGT approved this study.

Data were collected from each patient’s medical record every 3 months. The following baseline variables were collected at presentation: age, sex, race/ethnicity, duration of symptoms, diagnosis date, tumor location, and neuro-imaging characteristics. After surgical resection, we collected the following variables: resection date, extent of surgical resection, histopathologic diagnosis per the 2007 WHO classification of brain tumors,^32^ and treatment received. The extent of surgical resection was determined by postoperative magnetic resonance imaging using response assessment criteria in pediatric neuro-oncology^33^ and was categorized into the following groups: gross total resection (GTR), near-total resection, subtotal resection, and partial resection.^33^

### Clinical Outcomes

Clinical outcomes included death, tumor progression, and lost to follow-up. Progression was defined as radiographic (at least a 25% increase in two-dimensional measurements of the visible tumor(s) on imaging) and/or histopathologic evidence of disease recurrence, or progression after previously documented complete remission and/or stable disease. PFS was defined as the time that elapsed between treatment initiation and tumor progression. OS was calculated from the date of diagnosis to the date of last follow up, death by any cause, or lost to follow up if no vital status was known after the event. Data were analyzed in two eras—Era 1 (2010-2013) and Era 2 (2014-2017)—to compare outcomes before and after the CBNP was fully established (December 2013).

### Statistical Analysis

We performed descriptive statistics, used Mann-Whitney U and Fisher exact tests to determine the statistical significance of differences in continuous and categorical variables between subgroups, and performed a Cox proportional hazards regression model for continuous predictors (age at diagnosis and duration of symptoms). Variables included death, progression, and PFS and OS at 3 and 5 years, whereas age, sex, race/ethnicity, tumor type, duration of symptoms, presence of metastasis, and extent of surgical resection were covariates.

Kaplan-Meier survival curves were generated for PFS and OS, and we calculated hazard ratios and 95% CIs. Log-rank tests were used to determine the statistical significance of differences in PFS and OS. Statistical analyses were conducted using R software (3.5.0).^34^ We considered a *P* value < .05 to indicate statistical significance.

## RESULTS

### Patient Evaluation and Transfer Workflow

Children with a new diagnosis of CNST at HGT were discussed and images were shared via secure Web-platform with the RCHSD neuro-oncology team. Upon arrival at RCHSD, patients were assessed by the neuro-oncology team and admitted for surgery after obtaining relevant neuro-imaging. Postoperative care included a pediatric intensive care unit stay at RCHSD. Upon stabilization, the patient returned to HGT for additional management, which included chemotherapy, supportive care, radiotherapy, rehabilitation, surveillance, and neuro-imaging follow up. After histopathology reports were completed at RCHSD, postoperative treatment plans were discussed and designed between the teams at RCHSD and HGT. Cross-border communication between teams, including review of postoperative and follow-up images, and additional treatment recommendations, was conducted via teleconference and secure e-mail exchange. Additional information is provided in Figure 2 and the Appendix.

### Capacity Building: Targeted Training and Infrastructure Enhancement

To ensure high-level care before and after surgical resection, targeted neuro-oncology training (in person and virtual) was provided to the medical team at HGT, including pediatric oncologists (n = 4), pediatricians (n = 8), nurses (n = 32), and ancillary staff (n = 12). One initial challenge was the scarce number of pediatric oncologists who were trained to care for children with CNSTs. An advanced practice provider model was used as a solution for this shortage in which eight pediatricians received specialized training in neuro-oncology and pediatric intensive care. They now provide specialized care to critically ill patients in a dedicated pediatric intensive care oncology unit when patients with CNSTs are transferred back to HGT from RCHSD after their neurosurgical resection.

A structured plan to enhance infrastructure and provide access to medications, supplies, chemotherapy, radiology, and radiotherapy services was implemented, ensuring high-quality care for patients returning to HGT for postoperative care. Chemotherapy protocols were collaboratively developed by RCHSD and HGT teams on the basis of published recommendations for LMICs and were tailored to the local resources and availability of chemotherapy agents.^11,23,35-37^ Surgery services at RCHSD were provided as in kind. HGT leadership and the local foundation, Patronato HGT, committed to fund personnel, equipment, medications, supplies, and operational costs.

### Patient Clinical Outcomes

A total of 60 Mexican patients at HGT were diagnosed with CNSTs during the study period, and 49 underwent surgery at RCHSD. Patient characteristics are listed in Table 2.

**TABLE 2:**
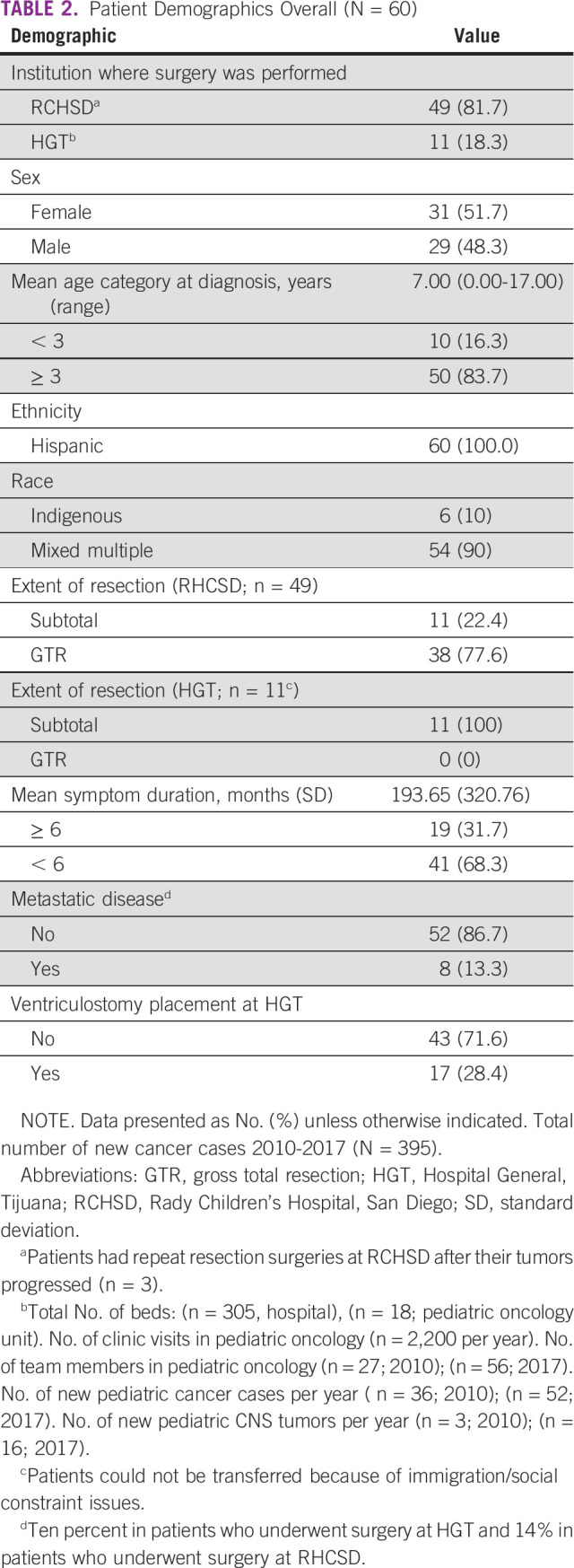
Patient Demographics Overall (N = 60)

The most common diagnoses were low-grade glioma (n = 15; 24.5%), medulloblastoma (n = 13; 22.4%), ependymoma (n = 8; 13.3%), craniopharyngioma (n = 6; 10%), and other (n = 18; 28.6%). The most common clinical features at presentation were emesis (53%), headache (51%), balance disturbances (25%), visual impairment (22%), papilledema (8%), seizures (8%), and vertigo (7%). The majority of patients had symptoms for more than 6 months (n = 34; 68.3%), did not have metastasis at presentation (n = 52; 86.7%), and did not have presurgical complications, such as respiratory or metabolic aberrations (n = 51; 85.7%). Seventeen patients (28.4%) had a ventriculostomy placement at HGT before transfer to RCHSD. Of note, none of the patients’ parents requested to continue care at RCHSD after surgery, and expressed that they were very satisfied with the follow-up care at HGT when surveyed upon their return to HGT.

### Extent of Resection and Survival Outcomes

The majority (n = 38; 77.6%) of patients who achieved GTR had surgery performed at RCHSD. Having surgery done at RCHSD, compared with HGT, was a significant determinant of GTR (*P* < .001). None of the patients who underwent surgery at HGT (n = 11) achieved GTR (Table 3).

**TABLE 3:**
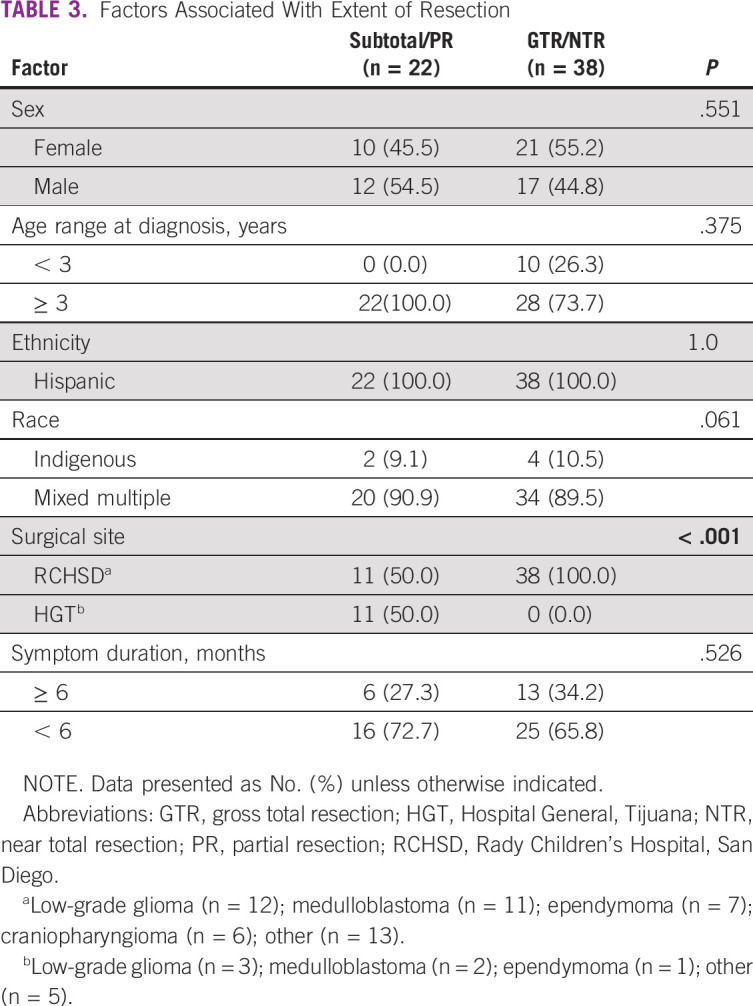
Factors Associated With Extent of Resection

**FIG 3:**
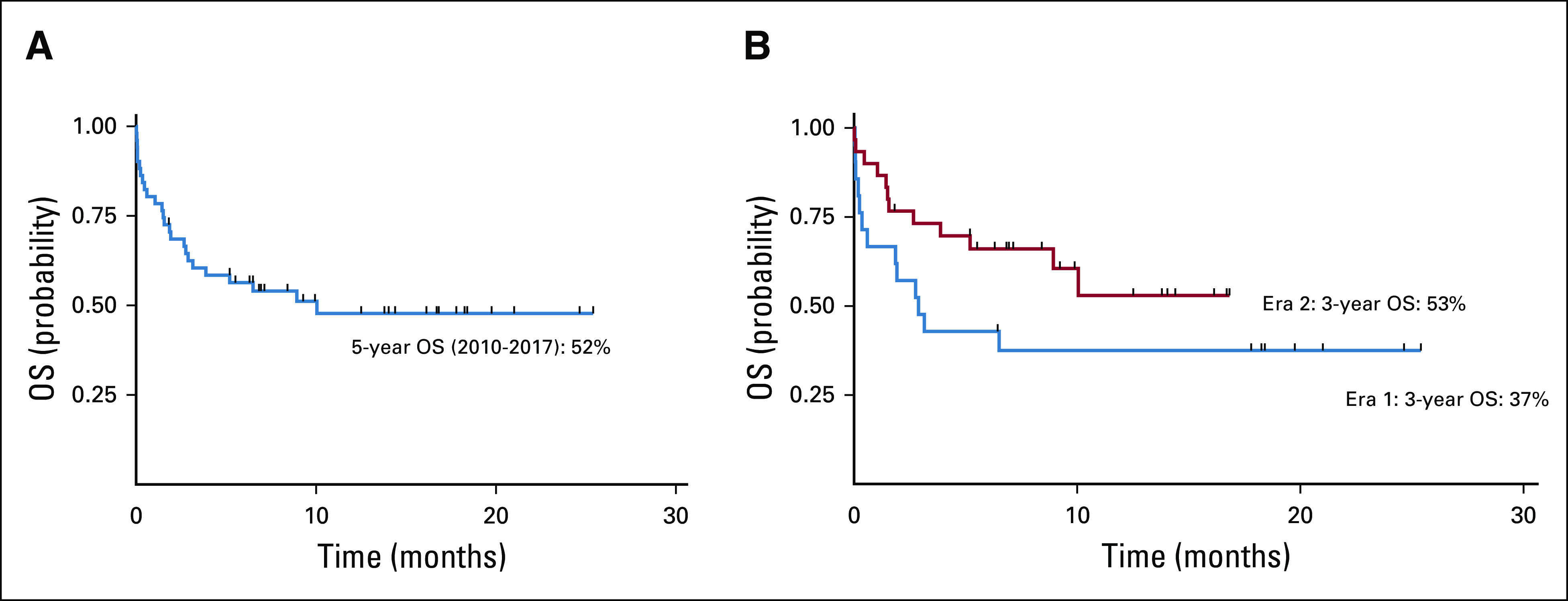
Kaplan-Meier survival curves for 5-year overall survival (OS) in the Cross-Border Neuro-Oncology Program. (A) Five-year OS (2010-2017): All CNS tumors (CNSTs), 52%; medulloblastoma, 40%; ependymoma, 44%; low-grade glioma, 91%; craniopharyngioma, 75%; other, 25%. (B) Three-year overall survival by Era (Era 1: 2010-2013; Era 2: 2014-2017). Era 1 3-year OS: 37%; Era 2 3-year OS: 53% (*P* = .23). Three-year OS (2010-2017): All CNSTs, 60%; medulloblastoma, 55%; ependymoma, 67%; low-grade glioma, 91%; craniopharyngioma, 75%; other, 25%.

GTR was a significant predictor of OS (hazard ratio, 0.250; 95% CI, 0.067 to 0.934; *P* = .026), and symptom duration of ≥ 6 months at initial presentation was a significant predictor of decreased PFS (hazard ratio, 3.576; 95% CI, 1.013 to 12.617). Age and race were not predictors of PFS or OS. Five-year OS improved from 0% before 2010 to 52% in 2017 (Fig 3A). Survival by diagnosis is shown in Figure 3. There was an incremental improvement in 3-year OS, from 37% at the end of Era 1 (2010-2013) to 53% in Era 2 (2014-2017; *P* = .023; Fig 3B and Table 4).

**TABLE 4:**
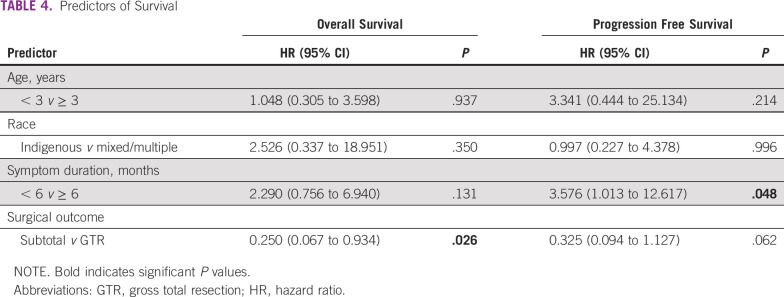
Predictors of Survival

## DISCUSSION

The CBNP led to dramatic improvements in the quality of pediatric neuro-oncology care for children with CNSTs diagnosed at HGT, including access to sophisticated neurosurgical management and increased survival.

Most global neuro-oncology efforts have focused on surgical camps to provide charity neurosurgical services.^26^ Our model leveraged the already-established pediatric oncology twinning program at HGT,^12,13^ responded to the 2015 Lancet Commission in Global Surgery,^28^ and focused on local health system strengthening, targeted training, capacity building, and advocacy.^12,13,25^ Faced with a neurosurgeon and neuro-oncologist ratio gap in Tijuana that was impossible to close expeditiously, as well as the imminent need to treat patients with CNSTs, the CBNP was a logical, temporary solution that capitalized on the close proximity between RCHSD and HGT across the United States-Mexico border.

Capacity building resulted in the implementation of disease-specific treatment guidelines and in a highly trained team able to provide high-quality intensive care expeditiously. Moreover, delays to timely neuro-imaging, diagnosis, neurosurgery, and radiotherapy have overall decreased. Communication and integration among health care teams has also improved. As reported by Qaddoumi et al,^22^ in addition to case discussions via online meetings, regular e-mails contributed to enhanced trust by the local team and helped reinforce the concepts discussed during telemedicine sessions.

Whereas published data on childhood cancer epidemiology in Mexico are relatively nascent, a recent report suggests that the estimated incidence of childhood cancer is 156.9 cases per 1 million per year and seems to be continuing to increase annually.^38^ CNSTs (9.1%) are the third most common pediatric cancer after leukemias (49.8%) and lymphomas (9.9%), and the most common cause of death in children age 5 to 14 years.^38^ These statistics are consistent with those reported at HGT in our study, where 12% of pediatric oncology patients between 2010-2017 were diagnosed with CNSTs. The diagnosis make-up in our cohort is consistent with the findings of other publications in HICs and LMICs.^1,4,6,30^

Five-year OS for all CNSTs at HGT improved from 0% (data from HGT hospital-based cancer registry, unpublished) before 2010 to 52% in 2017 after the implementation of the CBNP. There was a significant incremental improvement of 16% in 3-year OS from Era 1 (2010-2013) to Era 2 (2014-2017), highlighting the escalating positive impact of the CBNP. This increase is consistent with reports from other LMICs when long-standing twinning programs are implemented.^23,24^

GTR has an important role in improving the prognosis of patients with certain CNSTs, particularly medulloblastoma and low-grade glioma.^7,11,37,39-42^ Only children who underwent resection at RCHSD achieved GTR compared with patients who underwent surgery exclusively at HGT. This highlights the critical role of surgical expertise and adequate infrastructure and equipment to effectively cure CNSTs.^2,11,26,27,43^ Lower rates of GTR may reflect gaps in neurosurgery expertise and infrastructure in Tijuana and in similar settings in Mexico. Moreover, GTR was associated with better OS, which is consistent with the literature across many CNST subtypes,^39-45^ and supports the importance of GTR whenever possible.^1,4,8,23,46,47^ LMICs have not benefited from global advancements in neurosurgery, with most having minimal or no neurosurgical capacity.^26^ Moreover, there is a dramatic disparity in access to trained pediatric neurosurgeons in LMICs. There are an estimated 2,297 pediatric neurosurgeons in practice globally, with 85% operating in HICs, leaving only 350 pediatric neurosurgeons to care for a total population of 1.2 billion in LMICs with a ratio of one pediatric neurosurgeon per 3.6 million children.^48^

As reported in prior studies, in most CNSTs—other than certain medulloblastoma subgroups and certain low-grade gliomas^1,6^—patients with longer symptom duration at initial presentation had overall poorer outcomes. This suggests that timely diagnosis and swift cross-border transfer of pediatric patients with CNSTs to a site capable of providing vital, high-quality surgical resection afforded these patients the best chances of survival.

This collaboration was not unilateral as RCHSD benefited significantly from this partnership. Culturally appropriate strategies to ensure treatment compliance at RCHSD were adopted from HGT, specifically in Hispanic patients. In addition, pediatric oncologists at RCHSD are now more cognizant of resource conservation, as medications and personnel are often perceived as unlimited in the United States and these resources can be scarce in LMICs.

Our study must be considered in light of certain limitations. Our smaller-than-expected number of patients (based on the population size of Tijuana), related underdiagnosis of cases regionally, possible referral bias, and the evolving nature of the CBNP preclude an in-depth and consistent statistical analysis of patient outcomes over the study period. Moreover, assessing the outcome of patients who were transported to RCHSD is difficult without comparison with a control; however, denying children access to life-saving surgery for control purposes would be unethical. Instead, we may only compare outcomes with the small pool of patients who presented before the program was established. Furthermore, as a result of the lack of a well-established hospital-based registry (before the initiation of our program), specific data on imaging, diagnosis, neurosurgery, and radiotherapy delays were not systematically collected. Lastly, although the CBNP is an effective solution for a problem that was leading to the deaths of many children, we recognize that it is only a short-term step toward an established neuro-oncology infrastructure in Mexico, that it may impede the development of local neurosurgery services, and that the training of local pediatric neurosurgeons would be a long-term solution.

Many factors may hamper the structured growth of the CBNP in Baja California, including political and socioeconomic instability and a rapidly growing population. Ideally, a single regional center in an LMIC that is fully equipped and staffed could act as a referral center for pediatric neuro-oncology to improve the level of care.^47,49^

Future goals involve (1) growing philanthropic support for the CBNP (2) expanding access for the increasing number of patients and transitioning all facets of neuro-oncology care to HGT, (3) expanding cross-border collaboration among anesthesiologists and rehabilitation physicians, and (4) training local pathologists in neuropathology and a local neurosurgeon by instituting a neurosurgery residency program in Tijuana. Long-term sustainability requires cross-border commitment from many stakeholders and consideration of the local sociocultural perspectives. Global neuro-oncology programs should balance local challenges and opportunities and engage in capacity building through the development of training programs and formalized neuro-oncology and neurosurgery skills transfer to health care professionals in LMICs. This approach will advance their ability to effectively care for underserved children with CNSTs. Long-lasting improvements in disparate outcomes in LMICs will require cohesive health system planning with multiple stakeholders and establishing partnerships between institutions at HICs and LIMCs aimed at developing large-scale collaborative projects and research that have the potential to change national and international policy.

In conclusion, through bidirectional collaboration between cross-border communities and stakeholders, the creation of programs like the CBNP is feasible and may lead to significantly improved survival in underserved children with CNSTs. There are few twinning initiatives in pediatric neuro-oncology, and the CBNP has engendered an open and bilateral exchange of resources, expertise, and cross-border access. A health systems–strengthening approach facilitated interdisciplinary collaboration, enhanced infrastructure, and allowed for local capacity building. The CBNP offers opportunities for replication in HIC institutions in close proximity to LMICs across the United States-Mexico border and throughout the world.

## Appendix

### Patient Evaluation and Transfer Workflow

Children with a new diagnosis of CNS tumors at Hospital General, Tijuana (HGT), were initially assessed by the local pediatric oncologist. Each case was discussed and images were shared via secure Web platform with the pediatric neuro-oncology team at Rady Children’s Hospital, San Diego (RCHSD).

If the patient had neuroradiographic evidence of a tumor that was amenable to resection, after discussions between the neuro-oncology and neuro-surgery teams, and there was hospital administration approval, cross-border transportation was arranged by ambulance. Patients were considered eligible if they were age 0-21 years, were deemed appropriate for neuro-surgical resection, did not have prior surgery outside of RCHSD, were approved for transfer by US immigration, and did not have any social constraints, such as a caregiver unable to cross to San Diego because of family demands in Tijuana, a legal guardian unable to accompany patient because they did not reside in Tijuana, or a caregiver with health/mental conditions, among others.

This complex coordination involved negotiations with various stakeholders at both hospitals, including physicians, nurses, ambulance operators, administrative staff, hospital leadership, and at the United States-Mexico port of entry and US Consulate in Tijuana.

Upon arrival at RCHSD, patients were assessed by the neuro-oncology team and admitted for surgery after obtaining relevant neuro-imaging. Postoperative care included a pediatric intensive care unit stay at RCHSD. Upon stabilization, the patient returned to HGT via ambulance for additional treatment, including chemotherapy, supportive care, radiotherapy, rehabilitation, surveillance, and neuro-imaging follow up. After histopathology reports were completed at RCHSD, postoperative treatment plans were discussed and designed between the medical teams at RCHSD and HGT. All pathology services were completed at RCHSD, and medulloblastoma cases were subgrouped according to immunohistochemistry. Future planning will include using molecular studies to further enhance CNS tumor diagnostics. Pediatric oncologists at HGT had access to members of the neuro-oncology team at RCHSD for questions and advice. A bilingual (English/Spanish), bicultural (Anglo/Hispanic) pediatric oncologist (P.A.) at RCHSD was appointed the Cross-Border Neuro-Oncology Program medical director and coordinated care. Cross-border communication between teams, including review of postoperative and follow-up images and additional treatment recommendations, was provided to the HGT medical team via teleconference and secure e-mail exchange.
